# Placental Adaptations in Growth Restriction

**DOI:** 10.3390/nu7010360

**Published:** 2015-01-08

**Authors:** Song Zhang, Timothy R.H. Regnault, Paige L. Barker, Kimberley J. Botting, Isabella C. McMillen, Christine M. McMillan, Claire T. Roberts, Janna L. Morrison

**Affiliations:** 1 Early Origins of Adult Health Research Group, Sansom Institute for Health Research, University of South Australia, Adelaide, SA 5001, Australia; E-Mails: song.zhang@unisa.edu.au (S.Z.); barpl002@mymail.unisa.edu.au (P.L.B.); kb555@cam.ac.uk (K.J.B.); caroline.mcMillen@newcastle.edu.au (I.C.M); mcmcm003@mymail.unisa.edu.au (C.M.M.); 2 Departments of Obstetrics and Gynecology, University of Western Ontario, London, ON N6A 5C1, Canada; E-Mail: tim.regnault@uwo.ca; 3 Departments of Physiology and Biochemistry, University of Western Ontario, London, ON N6A 5C1, Canada; 4 The Robinson Research Institute, University of Adelaide, Adelaide, SA 5005, Australia; E-Mail: claire.roberts@adelaide.edu.au

**Keywords:** placental morphology, vascularity, substrate transport, IUGR

## Abstract

The placenta is the primary interface between the fetus and mother and plays an important role in maintaining fetal development and growth by facilitating the transfer of substrates and participating in modulating the maternal immune response to prevent immunological rejection of the conceptus. The major substrates required for fetal growth include oxygen, glucose, amino acids and fatty acids, and their transport processes depend on morphological characteristics of the placenta, such as placental size, morphology, blood flow and vascularity. Other factors including insulin-like growth factors, apoptosis, autophagy and glucocorticoid exposure also affect placental growth and substrate transport capacity. Intrauterine growth restriction (IUGR) is often a consequence of insufficiency, and is associated with a high incidence of perinatal morbidity and mortality, as well as increased risk of cardiovascular and metabolic diseases in later life. Several different experimental methods have been used to induce placental insufficiency and IUGR in animal models and a range of factors that regulate placental growth and substrate transport capacity have been demonstrated. While no model system completely recapitulates human IUGR, these animal models allow us to carefully dissect cellular and molecular mechanisms to improve our understanding and facilitate development of therapeutic interventions.

## 1. Introduction

In eutherian mammals, the placenta is the primary interface between the fetus and mother. One of the main functions of the placenta is to deliver nutrients and oxygen to the fetus. Failure of the placenta to deliver an adequate supply of nutrients to the fetus is termed placental insufficiency and results in intrauterine growth restriction (IUGR), affecting up to 5%–10% of pregnancies in developed countries [1,2,3]. IUGR is also associated with a high incidence of perinatal morbidity and mortality and an increased risk of cardiovascular disease and type II diabetes in later life [4,5,6,7]. In this review, we will focus on placental structure and function, factors affecting placental nutrient transport capacity, animal models of IUGR and regulation of placental growth and substrate transport in the IUGR pregnancy.

## 2. Placental Structure and Function

In mammals, the placenta is the primary interface between the fetus and mother and plays an important role in maintaining fetal growth by performing several physiological functions, which, following birth, are controlled by the kidney, gastrointestinal tract, lungs and endocrine glands. The main functions of the placenta include modulation of the mother’s immune response to prevent immunological rejection termed tolerance, facilitating the exchange of respiratory gases, water, ions, nutrients and wastes between the maternal and fetal circulations, and producing and secreting hormones, cytokines and other signalling molecules required to maintain pregnancy and to ensure placental and fetal development and growth [8].

Maternal blood supply to the placenta is established at the end of the first trimester of human pregnancy, with maternal blood entering the intervillous space of the placenta from the transformed spiral arterioles for substrate transport to the fetus [9,10,11]. The barrier between the maternal and the fetal circulations in the human hemochorial placenta consists of three fetal cellular layers: (i) the syncytiotrophoblast, a multinucleated epithelial layer formed following the fusion of the mononucleated villous cytotrophoblasts; (ii) villous stromal tissue and (iii) the fetal capillary endothelium (Table 1) [12]. The syncytiotrophoblast has two polarized plasma membranes: the maternal-facing microvillous plasma membrane (MVM) and the basal membrane (BM). The syncytiotrophoblast is the main regulator of substrate exchange and nutrient transporters are expressed on both plasma membranes [13].

**Table 1:** An across species comparison of placental shape and structure [14,15,16].

Species	Placental Shape	Placental Structure
Humans	Discoid	Hemochorial
Ruminants (Sheep, cattle, goats)	Cotyledonary	Epitheliochorial
Rodents (rats, mice)	Discoid	Hemochorial
Pigs	Diffuse	Epitheliochorial
Horses	Diffuse	Epitheliochorial
Carnivores (cats, dogs)	Zonary	Epitheliochorial
Primates	Discoid	Hemochorial

It is important to understand the role of placental function in the IUGR fetus from a clinical and scientific point of view. However, *in vivo* studies of the human placenta are difficult as the methodology presents risks to both the mother and fetus. Therefore, the majority of the body of knowledge about the placenta and its function is a result of ultrasound studies across gestation or those performed in placentas collected at delivery or in animal models. In terms of morphological similarities, the placenta of higher primates is the most comparable to that of the human, with a discoid hemomonochorial structure, where fetal trophoblast cells are in direct contact with the maternal blood supply [15]. However, primates that are suitable for experimental studies are small, have small fetuses, and often abort or deliver prematurely following intrauterine surgery, limiting their use in placental studies [17]. Therefore placental nutrient transport and exchange has been extensively studied in the rabbit, guinea pig, rat, mice and human [18,19,20]. However, one of the most widely used animal models for studies of fetal development and placental function is the sheep [21,22,23].

One of the advantages of working with sheep compared to other animal models of pregnancy is that the functional responses of the sheep fetus to short or long periods of placental insufficiency can be studied *in utero* [24,25,26,27,28]. Like the human, organogenesis in the sheep occurs during early gestation with the functional maturation of the renal and cardiovascular systems by late gestation [23]. In addition, pregnant ewes are comparable to humans in size with equally large fetuses that tolerate intrauterine surgery well and most organ systems develop before birth [29]. Therefore, vascular catheters can be surgically implanted in the fetus, exteriorized through the ewes’ flank and maintained for several weeks, allowing for repetitive blood sampling from unanaesthetised pregnant ewes and their fetuses to measure blood gases, hormones and cardiovascular parameters in response to placental insufficiency during late gestation [23,30].

In sheep, implantation begins at ~14–16 days gestation (term = 150 ± 3 days gestation) and is complete by ~22 days gestation, marking the initiation of placentation [31], which is the process of placental growth and development, resulting in the maturation of functional units called placentomes. Sheep have a non-invasive, cotyledonary type of placentation in which specialized areas of the endometrium termed caruncles are attachment sites for trophoblasts of the chorion forming 50–90 placentomes during a normal singleton pregnancy (Table 1) [14,16]. Placentomes are divided into the maternal (caruncle) and fetal (cotyledon) portions and consist of interdigitated maternal crypts and fetal villi that develop through synchronised growth between the trophoblast and caruncular endometrium [16,32]. Morphologically, the maternal portion of the placentome is composed of maternal connective tissue, maternal capillaries and maternal epithelial cells; while the fetal portion of the placentome is composed of fetal trophoblasts, connective tissue and fetal capillaries. The placentomes grow rapidly following implantation and reach maximum weight at ~75–80 days gestation, from which point weight declines until term [33]. However, placentomes undergo progressive structural remodelling, with attenuation of all cell layers in both fetal and maternal tissues, bringing fetal and maternal capillaries in close proximity to allow increased capacity for substrate transfer during the second half of gestation when fetal demand for nutrients is high [16].

Placentomes are classified into four types (A–D) based on their gross morphology [34]. The fetal side of the placentome is defined as a thin hemophagous zone where maternal blood extravasates between the maternal crypts and fetal villi. Type A placentomes are rounder in shape and the hemophagous zone is inverted inside of the bulk of the placentome in which only a small area of the zone is visible externally. Type D placentomes are flatter in shape and the hemophagous zone is everted and covers the entire fetal surface of the placentome. Type B and C placentomes are intermediate in the degree of hemophagous zone eversion that is present. Throughout gestation, type A and B placentomes dominate, accounting for ~60% of the total number in a normal pregnancy. Type C and D placentomes are less common, occurring with greater frequency in multiple pregnancies and during late gestation. They are larger, heavier and more everted compared to type A placentomes. Although, the placentome types have different structures, it is not known if they have differential functions [32]. Previous studies have suggested that placentome eversion is an adaptation that occurs to increase the efficiency of placental nutrient transfer to the fetus [35]. In sheep, gross placentome morphology changes progressively throughout gestation with an increase in the number of everted placentomes, type C and D, in late gestation at 125–135 days [35,36]. Interestingly, it has been reported that early exposure to high plasma cortisol concentrations decreases the proportion of type C and D placentomes, which indicates that developmental shifts in placentome classification are not restricted to eversion [16].

## 3. Factors Affecting Placental Substrate Transport Capacity

Birth weight depends not only on maternal nutrition but also on the placenta’s ability to transport substrates from the maternal to the fetal circulation [10]. Placental efficiency or fetal-placental weight ratio, defined as grams of fetus per gram of placenta, can vary between species and pregnancies [37]. At any gestational age, placental efficiency measurements provide an indication of the conditions experienced *in utero* and the extent to which placental adaptations during intrauterine development have occurred in order to meet fetal growth demands. These morphological and/or functional adaptations determine placental substrate transport capacity and efficiency to the fetus. The major substrates required for fetal growth include oxygen, glucose, amino acids and fatty acids [9,12,13].

While the placenta regulates the transport of nutrients to the fetus according to the mother’s ability to deliver them, fetal demands as well as hormones and growth factors secreted by the placenta, the weight, size and shape of the placenta also affect its ability to transfer nutrients [38,39,40]. The transfer of highly permeable molecules, such as gases, oxygen and carbon dioxide, is influenced by blood flow and occurs via simple diffusion, whereas less permeable substrates are transferred through passive and active transport processes. Glucose is transported across the placenta via facilitated diffusion and is orchestrated by hormones secreted by the placenta [11]. Therefore, net glucose transfer is highly dependent on the maternal-fetal concentration gradient. Nutrients such as amino acids are transported via active transport using the charge provided by sodium ions and those that are transported via exchange for another amino acid [13]. Furthermore, these processes also depend on morphological characteristics of the placenta, such as placental size, surface area for exchange, vascularity as well as blood flow [15].

### 3.1. Fetal Oxygenation

Simple molecules such as oxygen and carbon dioxide are transported by diffusion and bulk flow. Oxygen (O_2_) plays a critical role in the development and function of the placenta and fetal hypoxaemia is a common condition of complicated pregnancy. Oxygen diffuses from the maternal to the fetal circulation across a placental epithelial layer that consumes O_2_. This O_2_ consumption generates a transepithelial oxygen partial pressure (PO_2_) difference whose magnitude depends upon the rate of umbilical and uterine blood flow, fetal and maternal blood oxygen carrying capacity, haemoglobin oxygen binding affinity, placental surface area and placental permeability [41]. Placental gas exchange occurs across cellular layers between the uterine and fetal circulations, once vascular beds have matured to allow adequate supply across the two circulations [14]. As a result, placental oxygen levels are low in the first trimester, increasing significantly by the second trimester. Therefore the fetus develops in a hypoxic environment during early pregnancy until the utero-placental vasculature can provide efficient gas exchange. Currently, much of the understanding of fetal and placental respiration is from the study of sheep in late gestation [42]. As the placenta is a highly metabolically active organ, it consumes a large quantity of the oxygen taken up from maternal circulation; some 80% in mid-gestation [42] and 40%–60% in late gestation [43]. Moreover, despite decreases in maternal oxygenation and uterine blood flow, the PO_2_ gradient across the placenta remains constant in order to sustain fetal oxygen delivery at a normal rate [44].

### 3.2. Placental Size and Morphology

Placental size has a direct effect on the capacity for nutrient transfer via changes in surface area for transport. Placental weight is positively correlated with birth weight at term in many animal models [15]. In humans, placental weight progressively increases throughout pregnancy, while in rodents and sheep, placental weight increases before plateauing in mid to late gestation and then declines until term [45]. The small placenta increases nutrient transport capacity via morphological adaptations such as increased relative surface area for nutrient exchange, vascularity and decreased barrier thickness. These adaptations affect placental transport capacity and alter fetal to placental weight ratio.

### 3.3. Blood Flow and Vascularity

Blood flow is a major determinant of placental function and fetal growth. Vascularity of the sheep placenta increases in mid gestation, due to increases in the number and surface density of the placental capillaries, particularly those in the fetal side of the placentomes [46]. Vasculogenesis, formation of new blood vessels, and angiogenesis, formation of new branches from pre-existing vessels [47], are critical to form a vascular system needed for effective transport of nutrients, oxygen, and waste products. Several factors have been identified as important regulators of these processes, including the vascular endothelial growth factor (VEGF) family, basic fibroblast growth factor (bFGF), epidermal growth factor (EGF), platelet-derived growth factor (PDGF) and angiopoietin-1 and -2 [48]. The VEGF proteins and receptors VEGFR1 (FLT-1) and VEGFR2 (FLK-1) are the most studied family of growth factors known to regulate the processes of vasculogenesis and angiogenesis. In pregnancy, VEGF is expressed by human villous and extravillous trophoblasts and also participates in the proliferation, migration, and metabolic activity of trophoblasts [49]; bFGF acts as a modulator of tissue differentiation and placental angiogenesis [48]. Angiopoietin-1 and its antagonist angiopoietin-2 act on the angiopoietin receptor (TIE-2) to regulate vascular integrity and remodeling [50]. Angiopoietin-1 and -2 have both been detected in decidual and placental tissues [51]. Vascular growth is necessary to increase placental-fetal blood flow across gestation [52,53,54]. Therefore fetal growth is linked to the capacity of the placenta to supply oxygen and nutrients for transfer to the fetus [55].

### 3.4. Insulin-Like Growth Factors

Insulin-like growth factors (IGFs), IGF-1 and IGF-2, are single chained polypeptides that promote fetal and neonatal growth, acting in response to fetal, maternal and placental signals such as nutrients, oxygen and hormones via the IGF receptors, IGF-1R and IGF-2R, and the insulin receptor (InsR). IGF-2 is the most abundant of the fetal IGFs, however, deletion of either *Igf* gene results in reduced birth weight [55]. IGF-1 regulates fetal growth in response to nutrient availability while IGF-2 stimulates placental growth and differentiation [56]. Furthermore IGFs regulate substrate transport and hormone secretion and influence fetal growth indirectly by influencing maternal substrate availability or directly by influencing placental nutrient uptake and transport [56]. In human trophoblasts, IGF-1 and IGF-2 stimulate glucose and amino acid uptake [57]. Elevated maternal plasma IGF-1 has been shown to increase fetal glucose and amino acid uptake in the guinea pig in early pregnancy [27,28]. In sheep, elevated plasma IGF-1 concentrations in early pregnancy are associated with increased maternal plasma glucose concentrations and enhanced fetal growth [56]. Furthermore in late pregnancy, acute treatment with IGF-1 alters placental metabolic function and increases glucose delivery to the sheep fetus [58]. IGF-1 stimulates system A amino acid uptake in cultured trophoblast cells and can act by both the IGF-1R and InsR [57]. Interestingly, in a knockout mouse model, deletion of the gene transcript for placenta specific expression of *Igf2* results in the reduction of placental growth while fetal growth is maintained in association with an up-regulation of glucose transporter (GLUT3) and amino acid transporter (SNAT2) during mid gestation, however IUGR still develops near term [59,60,61].

### 3.5. Placental Apoptosis, Autophagy and Glucocorticoid Actions

The role of the apoptosis cascade has been characterized in human villous trophoblast turnover and syncytium formation [62]. Apoptosis occurs in placentas of normal human pregnancies, and is regulated by the effector caspase pathway and the apoptosis inhibitor B-cell lymphoma 2 (BCL-2) in the trophoblast [62].

Autophagy is a highly regulated and dynamic process involving invagination and degradation of cytoplasmic components that maintain cellular homeostasis and promote cell survival in response to environmental stresses such as starvation and hypoxia [63]. Autophagy-related proteins such as beclin-1, light chain 3 isoform B (LC3B), and damage-regulated autophagy modulator (DRAM) are present in the trophoblast of human placenta during early, mid and late gestation [64,65]. Beclin-1 is part of an early complex that promotes synthesis and growth of pre-autophagosomal membranes [66]. LC3B is synthesized as proLC3B and converted to LC3B-I by autophagy-related proteases, which is further processed into LC3B-II and integrated into membranes of autophagosomes upon induction of autophagy [66]. DRAM is a lysosomal protein that regulates autophagy in a p53-dependent manner [67].

*In utero*, the placenta and embryo/fetus are each exposed to physiological glucocorticoids arising from either the maternal or fetal adrenal glands. Glucocorticoid actions are moderated by the glucocorticoid receptor and two isoforms of intracellular 11β-hydroxysteroid dehydrogenases (11βHSDs) [68]. 11βHSD1, which is a reduced nicotinamide adenine dinucleotide phosphate-dependent isoform, acts to convert biologically inert cortisone to the active cortisol, whereas 11βHSD2, which is a unidirectional nicotinamide adenine dinucleotide-dependent enzyme, catalyzes the conversion of the biologically active cortisol to the inert cortisone. In placenta, 11βHSD1 protein is expressed specifically in the placental villous endothelial cells, amnion, chorionic and extravillous trophoblasts, while 11βHSD2 protein is localized exclusively in the syncytiotrophoblast and invasive extravillous trophoblasts with no expression in the chorion or amnion [69]. 11βHSD1 expression increases throughout pregnancy in response to progesterone in human placenta [70]. In human pregnancy, placental 11βHSD2 activity increases markedly in the third trimester of pregnancy at a time when maternal circulating levels of glucocorticoid are rising [71]. In contrast, the placental inactivation of cortisol via placental 11βHSD2 activity decreases in the latter stages of gestation in sheep and is not present in term ovine placenta [72]. The fetus is normally protected from the high levels of maternal cortisol by placental 11βHSD2 [69].

### 3.6. Placental Transporter Abundance

The placenta’s ability to transport substrates for fetal growth is also influenced by the abundance, activity and localization of transporters in the placental membranes [73]. Furthermore, transport of nutrients such as glucose and amino acids is also determined by the placental barrier and specifically the expression and activity of particular nutrient transport systems in the placental syncytiotrophoblast plasma membranes, MVM and BM [9]. Glucose and amino acid uptake into fetal circulation is dependent on three steps: (1) uptake from maternal circulation by transporters on the microvillous membrane of the syncytiotrophoblast; (2) transport across cytoplasm of the syncytiotrophoblast; and (3) transport across the fetal-facing basal membrane of the syncytiotrophoblast into fetal circulation [14]. Enhanced expression of nutrient transporters per unit of surface area is another key compensatory mechanism used to increase efficiency of the small placenta. Activity of placental glucose and amino acid transport systems is influenced by a wide range of environmental factors including heat stress, hypoxia, under- and overnutrition as well as exposure to placental hormones [55]. Placental glucose, amino acid and fatty acid transport systems are discussed in detail below.

#### 3.6.1. Placental Glucose Transport Systems

Glucose is the primary nutrient required for the fetus and placenta [13,74]. However, the fetus and placenta have a limited ability for producing glucose, therefore glucose availability is dependent on supply from the maternal circulation [13,75,76,77]. Placental glucose uptake and transport to the fetus occurs down the concentration gradient from maternal to fetal circulation where it crosses the placental barrier via facilitated transport [14,78]. This is mediated by a family of sodium-independent transporter proteins, encoded by 14 different genes of the SCL2A family, called glucose transporters (GLUTs) [78]. GLUTs are present on both plasma membranes of the syncytiotrophoblast [13]. As glucose is transported down the concentration gradient, higher maternal glucose concentrations compared to fetal glucose concentrations drive net glucose transport toward the fetus. A high density of GLUTs in the MVM, together with a large surface area, allows for rapid glucose uptake into the syncytiotrophoblast. This provides adequate glucose supply for placental consumption while also maintaining a gradient between the syncytiotrophoblast and fetal circulation, ensuring fetal supply [13]. GLUT expression on the BM is much lower than the MVM and is associated with a smaller placental surface area, which suggests transport across the BM is the rate-limiting step of placental glucose metabolism [9,13]. The sheep and human placenta have two primary glucose transporters including GLUT1 (SLC2A1) and GLUT3 (SLC2A3) and less abundant glucose transporters including GLUT4 (SLC2A4) and GLUT8 (SLC2A8).

GLUT1 is insulin independent and ubiquitously expressed in a variety of human tissues including muscle, adipose, brain and endothelial cells [74]. GLUT1 is the main glucose transporter in the placenta and is highly expressed on both the microvillous and basal membranes of the syncytiotrophoblast [13,79]. In humans, GLUT1 expression is higher in the MVM compared to the BM, allowing for increased glucose uptake from maternal circulation [9,80]. In sheep, GLUT1 is localised to the base of the syncytial layer of the placenta, which is derived from the maternal epithelial cells and chorionic binucleated cells, and the baso-lateral surface of the trophoblast layer [81]. However, GLUT1 has not been found on the interdigitated microvilli of the trophoblast and syncytial layer [14]. In sheep, placental GLUT1 mRNA expression increases throughout gestation, peaking at 120 days in singleton pregnancies and 140 days in twins [79].

GLUT3 is insulin independent and its expression is cell-specific, depending on the stage of pregnancy [78]. In both humans and sheep, GLUT3 is expressed throughout gestation but decreases toward term, and therefore plays a greater role in the transport of glucose during early fetal development [74,78]. Furthermore, GLUT3 expression in the placenta has been detected in the cytotrophoblast but not the syncytiotrophoblast in the first trimester of human pregnancy [78]. However, in the third trimester placental GLUT3 expression is localised to the vascular endothelial cells of the fetal blood vessels and stromal cells [14,78,80]. In sheep, GLUT3 has been found on the microvillous junction between the syncytium and trophoblast layer [81]. Interestingly, GLUT3 is only expressed in one other tissue aside from the placenta, the brain [74,80]. The brain, like the fetus, depends on a constant supply of glucose in order to sustain energy production. Therefore, expression of GLUT3 in the placenta may maintain glucose supply to the fetus, even when maternal plasma glucose concentrations are low [14]. Furthermore, GLUT3 has a higher affinity for glucose than GLUT1 and is therefore more efficient in transporting glucose at low concentrations [81]. The high affinity of GLUT3 for glucose means that in conditions where maternal glucose concentrations are lower than normal, GLUT3 may be the key transporter in tissues where glucose is the primary metabolic substrate [74].

GLUT4 is insulin dependent and its immunostaining was detected in fibroblasts from amnion and chorion [82]. A strong GLUT4 signal was also observed in intravillous stromal cells, appearing to co-localize with InsR [83]. GLUT8 was localized to the chorionic epithelial layer and uterine epithelial cell from mid to late gestation and its expression in placenta increased during late gestation [84,85].

#### 3.6.2. Placental Amino Acid Transport Systems

The human placenta expresses more than 20 different amino acid transporters [13]. Fetal amino acid concentrations are generally higher than maternal amino acid concentrations, reflecting an active transport mechanism across the placenta [13,86]. However, the placenta not only transports amino acids from mother to fetus but also produces and utilizes amino acids to meet its own metabolic need, and therefore plays a key role in determining the flux of amino acids into the fetal circulation [9]. Many types of placental amino acid transporters have been identified and are characterized into distinct systems [13,86]. There are several major classes of amino acid transporters present in the placenta, including neutral amino acid transporters, cationic amino acid transporters and anionic amino acid transporters [87,88].

System A amino acid transporters are an example of neutral amino acid transporters that facilitate the uptake of small non-essential neutral amino acids such as alanine, glycine and serine. Uptake occurs against their concentration gradient simultaneously with the transport of extracellular sodium into the cell. While system A transporters are present on both placental membranes, expression is greater on the MVM. System A activity contributes to the high intracellular concentration of amino acids such as glycine, which is exchanged for extracellular essential amino acids by system L transporters [9]. Therefore, system A transporters are important for the placental transport of both non-essential and essential amino acids [13]. System A consists of three sodium-coupled neutral amino acid transporter (SNAT) proteins, each encoded by independently regulated genes: SNAT1 (SLC38A1), SNAT2 (SLC38A2) and SNAT4 (SLC38A4) [59,89]. SNAT2 is ubiquitously expressed in mammalian tissues, while SNAT1 is predominately expressed in the brain, heart and placenta and SNAT4 expressed in the liver and placenta [89]. Throughout gestation, the activity of system A transporter increases, although the individual contributions of the three SNATs to total system A activity varies [13]. SNAT1 and 4 are found on both the MVM and the BM of the placenta [90]. SNAT1 has been shown to play a significant role in the human term placenta [91]. SNAT4, which has a lower substrate affinity for neutral amino acids, has a higher contribution in the first trimester placenta than the term placenta [89].

System L transporters are sodium independent exchangers of neutral amino acids. Non-essential amino acids are exchanged for essential amino acids with aromatic or branched side chains such as leucine or phenylalanine to allow for transport against their concentration gradient [92]. System L transporters consist of a heterodimer formed from a light chain protein, large neutral amino acid transporter (LAT)1 (SLC7A5) or LAT2 (SLC7A8), and a heavy chain transmembrane protein: 4F2hc/CD98 (SLC3A2) [92,93]. 4F2hc and LAT1 expression is higher in term than mid gestation placentas [93]. Localization of the LAT isoforms are polarized, with LAT1 predominately found on the MVM and LAT2 on the BM [94], although LAT2 has also been shown to have functional activity in the MVM [95]. However, localization of LAT2 on the BM allows for the exchange of amino acids between non-essential amino acids in the fetal circulation and essential amino acids in the syncytiotrophoblast cytoplasm [96]. Two additional system L light chains, LAT3 and LAT4 have recently been reported in placental tissues [97,98]. They are localized to the BM and may play an important role in the net efflux of amino acids to the fetus [99]. While transporting a restricted group of system L substrates (leucine, isoleucine and phenylalanine), LAT3 and LAT4 also appear to differ from the traditional system L transport in that they do not require the co-expression of 4F2hc and function as facilitative diffusion transporters [99,100].

It is well established that the cationic amino acid transporter (CAT) is the main transporter for cationic amino acids in MVM [101]. Kamath *et al.* also found that CAT1 (SLC7A1), CAT2 (SLC7A2), and CAT4 (SLC7A4) are expressed in cultured trophoblast cells and in BeWo choriocarcinoma cells [102], supporting the possibility that multiple members of the CAT family are present in BM of the placenta. CAT is a sodium independent electrogenic transporter, which interacts only weakly with neutral amino acids and therefore is specific for cationic amino acids.

Anionic or system X_AG_ amino acid transporters are capable of sodium dependent and d-aspartate-inhibitable glutamate/aspartate transport activity. A family of five anionic amino acid transporters (excitatory amino acid transporters EAAT1-5) have been cloned and, of these, EAAT1-4 are expressed in the human and rat placenta [103,104,105]. EAAT1-4 are present in both plasma membranes of the syncytiotrophoblast and increase over the last trimester [105,106].

#### 3.6.3. Placental Fatty Acid Transporter Systems

Fatty acids are important precursors of bioactive molecules, which are structural components of cells and provide a major source of energy [13]. Two sources of fatty acids are taken up by the placenta from the maternal circulation, the esterified fatty acids that are present in triglycerides (TG) and the nonesterified fatty acids (NEFA) [13]. The primary source of fatty acids taken up by the placenta is maternal TG because their uptake increases in the last trimester compared with NEFAs [107].

Fatty acid transfer from the mother to the fetus can be accomplished via simple diffusion, as with NEFAs [107]. However, in late gestation the rate of simple diffusion may not be adequate to supply the fetus [13,108]. Kazantzis and Stahl stated that the cellular membrane fatty acid transport proteins (FATPs) are important for the cellular uptake of long chain fatty acids [108]. There are five transport proteins in humans, including FATP1-4 and FATP6 [109]. FATP1 is present on both the MVM and the BM, but the exact cellular location of the others remains unknown [110]. Fatty acid translocase (CD36) is the membrane-associated protein expressed in placenta with the ability to transport fatty acids [110]. Upon entering the syncytiotrophoblast the hydrophobic fatty acids are transported across the cytosol to the BM or to other sites within the cell where they undergo esterification or beta oxidation. To accomplish this they bind with fatty acid binding proteins (FABPs) [13]. There are four forms of the fatty acid binding proteins in the human placenta, including FABP1 and FABP3-5 [111].

We have summarized the major placental changes in morphology and substrate transport capacity that regulate placental efficiency and fetal growth in normal pregnancies. As we know, placental insufficiency results in a failure of the placenta to deliver an adequate supply of substrates to the fetus and IUGR develops. Therefore, several different experimental methods used to produce placental insufficiency and induce IUGR in animal models and a range of factors that regulate placental growth and substrate transport capacity have been discussed in human and animal IUGR studies below.

## 4. The Placenta and Development of Intrauterine Growth Restriction

Intrauterine growth restriction is clinically defined as a birth weight below the tenth centile for gestational age where the fetus does not meet its growth potential [23,112]. IUGR affects 6% of Australian pregnancies [1] and is associated with a high incidence of perinatal morbidity and mortality [7]. IUGR neonates have a greater risk of hypoxic-ischaemic encephalopathy, intraventicular haemorrhage and necrotizing enterocolitis with longer hospital stays and higher health care costs [23]. Furthermore, epidemiological, clinical and experimental studies from around the world highlight an association between a poor intrauterine environment and poor health outcomes in adulthood [7,113,114,115]. In particular, these studies have demonstrated a significant relationship between small size at birth and the risk of developing coronary heart disease, hypertension and type 2 diabetes in adulthood [4,5,6]. Causes of IUGR can be of maternal, fetal or placental origin, including maternal smoking, alcohol and drug abuse, genetic defects, chromosomal abnormalities or placental pathologies [116]. In the developing world, IUGR is likely to be a consequence of poor maternal nutritional status prior to or during pregnancy whereas, in the developed world IUGR is commonly a consequence of placental insufficiency [117].

Several different experimental methods have been used to produce placental insufficiency and induce IUGR in both small and large animal models, and previously discussed in detail [23]. For example, methodologies include reduction of uterine blood flow by vascular occlusion or ligation in rats, guinea pigs and sheep, hyperthermia, placental infarction by repetitive embolization in pregnant ewes or uterine carunclectomy of non-pregnant ewes [23]. As sheep are used in all of these methodologies to induce IUGR, the sheep models of placental insufficiency are summarized below (Table 2).

**Table 2:** Summary of experimental models of placental insufficiency and their impact on the placenta and the fetus.

Experimental Intervention		Impact on the Placenta	Impact on the Fetus
**Surgical Umbilical Artery Ligation (SUAL)**	Isolation and ligation of one umbilical artery close to the fetal abdomen	placental infarction causing ↓ umbilical blood flow and ↓ placental substrate transfer [118,119]	Hypoxemia IUGR
**Maternal Hyperthermia**	Exposure of pregnant ewes to an environment with an increased ambient temperature	↓ uterine artery flow and ↓ placental weight due to ↑ maternal temperature [2,30]	IUGR
**Placental Embolism**	Repeated injection of microspheres (15μm) into the placenta via the umbilical artery through a catheter implanted in the descending aorta or fetal umbilical vein	block placental capillaries causing ↓ placental surface area [120]	Hypoxemia Hypoglycemia IUGR
**Uterine Carunclectomy**	Surgical removal of the majority of the endometrial caruncles from the uterus of non-pregnant ewes prior to conception	↓ placental weight due to ↓ placentomes [116]	Hypoxemia Hypoglycemia IUGR

## 5. Animal Models of Placental Insufficiency

### 5.1. Single Umbilical Artery Ligation (SUAL)

Single umbilical artery ligation (SUAL) involves the isolation and ligation of one umbilical artery close to the fetal abdomen [118]. SUAL causes a partial infarction of the placenta, which reduces umbilical blood flow and causes a reduction in the capacity of the placenta to transfer substrates and induces IUGR in the sheep fetus [118,119]. Furthermore, SUAL fetuses are significantly smaller than control fetuses from 117 days gestation and are chronically hypoxaemic (PO_2_ < 17 mmHg), but not acidotic in late gestation [121].

### 5.2. Maternal Hyperthermia

The hyperthermia-induced model of placental insufficiency induces fetal growth restriction by exposing pregnant ewes to an environment with an increased ambient temperature with a diurnal cycle of 40 °C for 12 h and 35 °C for 12 h, from ~38 days gestation until ewes are sacrificed at post mortem [2,122]. Ewes can be exposed to this treatment for ~17 days (early gestation), ~52 days (mid gestation) or ~96 ± 5 days (late gestation). This hyperthermia treatment results in an increase in maternal core body temperature from ~39 °C to 40 °C in hyperthermia pregnant ewes [53,123]. It also results in a redistribution of blood flow toward the peripheral vascular system, leading to a reduction in uterine and umbilical artery blood flow and a decrease in placental weight and IUGR [2,30].

### 5.3. Placental Embolism

Placental embolism of the placental vasculature aims to mimic the onset of IUGR in late gestation. This procedure involves repeated injection of microspheres (15 μm) into the placenta via the umbilical artery through a catheter implanted in the descending aorta or fetal umbilical vein [124,125,126]. The microspheres block the placental capillaries resulting in reduced surface area for the transfer of substrates from mother to fetus [120]. Placental embolism in late gestation causes acute decreases in placental substrate supply after each daily injection over a period of 10–20 days, which leads to chronic fetal hypoxemia, hypoglycemia and IUGR.

### 5.4. Endometrial Carunclectomy

Uterine carunclectomy limits the number of placentomes after surgical removal of the majority of the endometrial caruncles from the uterus of nonpregnant ewes prior to conception. This restricts the number of placentomes that form during pregnancy, consequently limiting placental and fetal growth [116]. Experimental restriction of placental growth results in fetuses that are chronically hypoxaemic and hypoglycemic [127]. Furthermore, the changes to nutrient supply and fetal blood gases in placentally restricted (PR) sheep fetuses are similar to those measured in cordocentesis studies of human infants who are born small for gestational age [116,121]. PR fetuses also have reduced placental and fetal weights at term [128].

## 6. Regulation of Placental Growth and Substrate Transport in IUGR Pregnancy

Fetal growth is dependent on substrate supply, which is dependent on substrate transport and its regulation. These processes also depend on morphological characteristics of the placenta, such as placental size, morphology, blood flow and vascularity. Therefore placental nutrient transfer capacity is specifically regulated by signals of fetal, maternal and placental origin in an effort to control fetal growth. However, in late gestation, when fetal nutrient demand is at its greatest, the compensatory upregulation is no longer sufficient to meet fetal demand and thus IUGR develops [129,130]. Here, we focus on the morphological and functional changes in the regulation of placental growth and substrate transport capacity in human pregnancy complicated with IUGR and animals models of placental insufficiency.

### 6.1. Placental Size and Morphology

In human pregnancies, Chen and coworkers have found a significant decrease in villi vascular density and cell proliferation in both trophoblast and stromal cell compartments within the IUGR placentas compared with control placentas at 25 to 41 weeks of gestation [131]. Other reports have shown a decrease in surface area, volume, and number of terminal villi, a reduced number of capillaries as well as an increased thickness of the exchange barrier in the stroma of IUGR placentas as compared with placentas from normal pregnancies [132,133]. Undernutrition in guinea pigs during pregnancy increases barrier thickness and reduces placental weight and placental area involved in nutrient exchange during late gestation [134]. In contrast, hypoxia during pregnancy cause a reduction in the barrier thickness between maternal and fetal circulation in the human and guinea pig placenta that has a direct effect on diffusional exchange [135,136]. In pregnant sheep, the total surface of the cotyledons and surface occupied by vasculature were greater at high altitude, whereas the number of cotyledons was smaller at high altitude [137]. These placental morphological changes may improve maternal and fetal exchange and display an efficient mechanism of adaptation to hypobaric hypoxia.

Experimental restriction of placental size by surgical removal of implantation sites prior to conception, multiple pregnancy or adverse conditions in early gestation restricts fetal growth, but increases fetal to placental weight ratio and placental efficiency in late pregnancy [15,53]. Conversely, an increased fetal to placental weight ratio may suggest fetal adaptations in response to the small placenta, which maximizes transplacental concentration gradients and/or partitioning of placentally derived nutrients to support and maintain fetal growth during suboptimal intrauterine conditions [55]. Adverse conditions, such as maternal undernutrition and hypoxemia resulted in increased eversion of placentomes and an increased proportion of type C and D placentomes in late gestation sheep fetuses [138,139]. In contrast, adverse intrauterine conditions induced by umbilical cord compression is associated with an increased proportion of type A and B, and fewer type C- and D placentomes during late gestation in sheep [140]. Therefore, adverse conditions in the intrauterine environment determine placental morphology during late gestation depending on the type and duration of the insult.

### 6.2. Oxygen Supply/Uptake, Blood Flow and Vascularity

In situations of placental insufficiency and IUGR, there appears to be an underdevelopment of the placental epithelial sites, with low hindrance to transplacental O_2_ diffusion reducing the surface area of exchange between the uterine and umbilical circulations, and resulting in decreased fetal umbilical oxygen supply [30,132]. Under hypoxic conditions, hypoxia inducible factor (HIF)-1, which consists of two subunits HIF-1α and HIF-1β, acts on the cell nucleus and regulates the expression of genes with hypoxia response element (HRE). HIFs recruit mechanisms to increase oxygen supply (erythropoiesis, angiogenesis, and vasodilation), decrease oxygen demand (increased glycolysis coupled with decreased oxidative metabolism), and regulate the cell cycle, apoptosis, and autophagy [141]. *In vitro* studies have demonstrated that hypoxia can affect the proliferation, differentiation, and invasion of cytotrophoblast cells [142]. Following a three day exposure to low oxygen, there was decreased HIF-1α and unchanged HIF-2α mRNA expression in cultured murine ectoplacental cones [143]. Hypoxia resulted in an increase in the transcription and translation of VEGF in cultured placental fibroblasts [144]. Low oxygen levels also resulted in a shift of the angiopoietin-1: angiopoietin-2 ratio in favor of angiopoietin-2, leading to vessel instability, angiogenesis and vessel remodeling [145].

Morphological studies show that physiological remodelling of the maternal uterine vasculature into spiral arteries is deficient in IUGR pregnancies due to inadequate trophoblast invasion [146,147]. This results in maternal blood entering the placenta at an abnormally high rate, which causes damage to the placental villi and may harm endocrine and transport functions of the placenta. The intensity of VEGF-A immunostaining in syncytiotrophoblast was significantly reduced in placental villous tissue from pregnancies complicated by IUGR and preeclampsia compared with the control group [148]. However, it has been shown that the expression of VEGF-A and bFGF was significantly higher in cytotrophoblasts, syncytiotrophoblasts, extravillous trophoblasts, vascular smooth muscle cells, chorionic villous stromal cells and villous vascular endothelial cells of the IUGR placenta when compared with those collected from normal-term pregnancies using semi-quantitative immunohistochemistry [149]. This suggests that these factors play a role in promoting increased endothelial cell proliferation, migration and pathological angiogenesis. Similarly in sheep, hyperthermia-induced placental restriction resulted in increased uterine blood flow and increased VEGF, angiopoietin-1, angiopoietin-2 and TIE-2 expression in the fetal portion (cotyledon) of the placentome in early gestation as well as reduced expression in FLT-1 and FLK-1 in the cotyledonary tissue in mid gestation, suggesting a disorganized fetal capillarisation and angiogenesis as well as compensatory transport capacity for the fetal circulation to uptake nutrients from the maternal circulation, which fails to maintain placental and fetal growth [53,54,123]. These alterations in angiogenic growth factors could impair normal placental vascular development and may contribute to the development of placental insufficiency, and ultimately intrauterine growth restriction.

### 6.3. IGFs

In knockout mice with a deletion of the placental-specific transcript (P0) of the *Igf2* gene, placental surface area is reduced and placental thickness increased, leading to reduced placental growth and fetal growth restriction [60,61]. Placental IGF-I mRNA expression was lower in the growth-restricted groups compared with normal pregnancies [150]. Similarly, the mRNA expression of IGF1, IGFBP1 and human placental growth hormone was significantly lower in placentas from human IUGR pregnancies compared with that in placentas of fetuses with normal growth [151]. However in sheep, caruncle IGF1 mRNA expression was increased at 90 days gestation in the placentas of hyperthermia-induced IUGR compared with the control group [152]. Cotyledon IGF2 and caruncle IGFBP4 mRNA expression were also elevated at 55 days gestation in these placentas [152]. In contrast, maternal hypoxia in mice from mid to late gestation resulted in a decrease in placental mRNA expression of IGF2, IGF1R and IGF2R [153].

### 6.4. Apoptosis, Autophagy and Glucocorticoid Action

Placentas from women with IUGR pregnancies show enhanced apoptosis when compared with those from normal pregnancy [154,155]. Cultured trophoblasts exposed to hypoxia alone show an upregulation of p53 activity and BCL-2-like protein 4 (BAX) expression and decreased expression of the anti-apoptotic BCL-2, all of which promote apoptosis [156]. Hypoxia/re-oxygenation results in even more marked apoptosis regulated by increased expression of the pro-apoptotic BAX and BCL-2 homologous antagonist (BAK) mRNA and protein and reduced expression of BCL-2 mRNA in human placental villous tissues [157]. Expression of p53 and the active form of caspase-3 is upregulated in villi from IUGR compared to control pregnancies, and the increase is predominantly in the villous trophoblast in humans [158,159]. The association between altered trophoblast cell turnover in IUGR and increased p53 expression was also shown to be reminiscent of that following exposure to hypoxia [160].

Compared with normal pregnant women, women with IUGR had increased placental levels of autophagy-related proteins including LC3B-II, beclin-1, DRAM, and p53 [161]. Furthermore, hypoxia induces apoptotic and autophagic changes in primary human cytotrophoblasts [162].

Placental 11βHSD2 activity was significantly reduced in deliveries complicated by IUGR compared with the term deliveries and with appropriately grown preterm deliveries, suggesting that glucocorticoids may, in part, contribute to impaired fetal growth closely controlled through placental 11βHSD2 expression [71]. There is evidence that exposure of the pregnant sheep to maternal undernutrition for a period extending beyond the preimplantation period, from 60 days before and the first 30 days after conception or from early to mid gestation (between 28 and 78 days gestation), results in a decrease in placental 11βHSD2 expression or activity [72,163]. In mice, maternal hypoxia during mid to late gestation resulted in a decrease in placental glucocorticoid receptor (GR) mRNA expression as well as placental 11βHSD2 mRNA and protein expression [153]. A previous study by Mericq *et al* found a lower expression and activity of 11βHSD1 in both chorionic and basal plates of the placentas from full term small for gestation age newborns compared with those in the placentas from appropriate for gestational age newborns, suggesting a possible compensatory mechanism to diminish the higher cortisol concentrations in fetuses with IUGR [164].

### 6.5. Glucose Transport Systems

Alterations in placental glucose transport have been implicated in adverse perinatal conditions such as IUGR and fetal hypoxia [165]. As glucose transport is dependent on the glucose concentration gradient from mother to fetus, the IUGR pregnancy has increased the transplacental glucose gradient and glucose uptake across the placenta [10] and this represents an example of how the IUGR fetus adapts to restricted placental size. In carunclectomized ewes with smaller placentas and a larger proportions of type D placentomes, the rate of glucose transfer to the fetus per gram of placenta is greater than that in controls [166,167]. Exposure to hypoxic conditions resulted in an upregulation in GLUT1 protein abundance and trans-epithelial glucose transport in BeWo choriocarcinoma cells [168]. GLUT3 mRNA expression and protein abundance in the trophoblast on the maternal aspect of the placenta was increased in the full-term IUGR placenta compared with normal placenta [169]. Increased placental GLUT3 expression was associated with increased activation of placental HIF-1α, suggesting that hypoxia may play a role in the upregulation of GLUT3. However, placental GLUT1 mRNA expression was decreased in the maternal aspect of the IUGR placenta and GLUT4 mRNA expression was increased in the fetal aspect of the IUGR placenta when compared with the control pregnancies, although there was no difference in placental GLUT1 or GLUT4 protein abundance between the treatment groups [169] (Table 3). Similarly, placental GLUT1, but not GLUT3 immunostaining in the terminal villi of severe preeclampsia cases (both with and without IUGR) was significantly lower compared with the control group [170]. Chronic hypoxia *in vivo* with high altitude pregnancies also resulted in a decrease in GLUT1 expression in the BM but not MVM of the placenta, leading to reduced nutrient supply and fetal growth [171]. In mice, maternal hypoxia during mid to late gestation resulted in a decrease in placental GLUT1 mRNA and protein expression in female fetuses, but no change in placental GLUT3 mRNA expression in both female and male fetuses [153]. Another study demonstrated that GLUT1 protein abundance is not altered in IUGR babies when compared to those that are appropriate for gestational age (AGA) [172]. Placental GLUT8 mRNA expression and protein abundance was decreased in late gestation in a sheep model of IUGR caused by placental insufficiency, which may contribute in part to the placental glucose transport deficit that occurs in this model [84]. Taken together, these data along with amino acid and fatty acid transport systems mentioned below suggest that the regulation of nutrient transport in the IUGR pregnancies may depend on the timing and type of the insult, and be species-specific and different from *in vivo* and *in vitro* studies.

**Table 3:** Summary of human and sheep models of IUGR and their impact on oxygen transfer, placental glucose, amino acid and fatty acid transporters.

Models of IUGR	Impact on Glucose Transporters	Impact on Amino Acid Transporters	Impact on Fatty Acid Transporters
Human IUGR	↓ GLUT1 mRNA, ↔ GLUT1 protein [169] ↑ GLUT4 mRNA, ↔ GLUT1 protein [169] ↑ GLUT3 mRNA & protein [169] ↓ GLUT1 protein [171] ↓ GLUT1 protein ↔ GLUT3 protein [170] ↔ GLUT1 protein [172]	↓ System A transporter activity [173,174] ↓ SNAT2 mRNA & protein [175]	NS
Sheep			
Surgical Umbilical Artery Ligation (SUAL)	NS	NS	NS
Maternal Hyperthermia	↓ GLUT8 mRNA & protein	↑ LAT-1 & LAT-2 mRNA [19]	NS
Placental Embolism	NS	NS	NS
Uterine Carunclectomy	NS	NS	NS

NS, not studied.

### 6.6. Amino Acid Transport Systems

In the IUGR placenta the different amino acid transport systems are affected. Tracer studies of placental amino acid transport have demonstrated that there is a significant reduction in the transplacental flux/fetal turnover ratios of essential amino acids [176,177,178]. For example, reduced uptake of leucine and lysine was found in vesicles from human IUGR placenta compared with the controls, suggesting decreased activity of placental transporters for cationic and neutral amino acids [176]. Similarly, there was a reduction of maternal leucine flux into the placenta and fetus in the hyperthermia sheep model of IUGR [178]. However, studies of fetal plasma amino acid concentrations have produced inconclusive results. Although earlier reports showed decreased fetal plasma amino acid concentrations [178], subsequent human and animal studies showed maintained or increased amino acid concentrations [22,177,179]. In severely growth restricted sheep fetuses, where placental and fetal weights are reduced by 40%–60%, umbilical uptake of oxygen, glucose and essential amino acids is significantly reduced, whereas the fetal/maternal ratio of the amino acids that are transported from the placenta to fetus showed normal or elevated fetal concentrations compared to control fetuses [22,30]. A previous study has shown that transplacental and total placental clearance of a branched-chain amino acid analogue, the non-metabolisable neutral amino acid aminocyclopentane-l-carboxylic acid per 100 g placenta, were significantly reduced in a sheep model of hyperthermia-induced IUGR, suggesting impaired placental non-essential amino acid transport in the IUGR placenta [180].

In normal pregnancy, system A transporter activity was shown to be the highest in the smallest babies per milligram of microvillous protein [181]. In contrast, placental system A transporter activity was not only reduced in IUGR compared with normal pregnancies but also related to the severity of IUGR [173,174]. Placental SNAT2 mRNA expression and syncytiotrophoblast immunostaining were significantly decreased in IUGR placentas with reduced umbilical blood flows compared with those in control placentas [175]. Hypoxia also decreases placental system A transport and activity in full-term human trophoblasts [182]. In mice, maternal hypoxia during mid to late gestation resulted in an increase in placental GLUT1 mRNA expression in female fetuses, but no change in placental SNAT4 mRNA expression. There was also a decrease in placental SNAT2 mRNA expression in both female and male fetuses [153]. Interestingly, placental LAT-1 and LAT-2 mRNA expression was elevated in the moderately growth restricted sheep fetuses where placental and fetal weight was reduced by 25% compared with the control placenta [19].

### 6.7. Fatty Acid Transport Systems

Hypoxia enhances the expression of FABP1, -3, and -4 in term human trophoblasts, suggesting that FABPs play a role in fat accumulation in the hypoxic placenta [111]. Similarly, hypoxia also resulted in increased FATP2 expression and reduced FATP4 expression in cultured primary term human trophoblasts [183]. However, placental CD36 mRNA expression was unaltered in human pregnancy complicated with IUGR [184].

## 7. Conclusions

**Figure 1:**
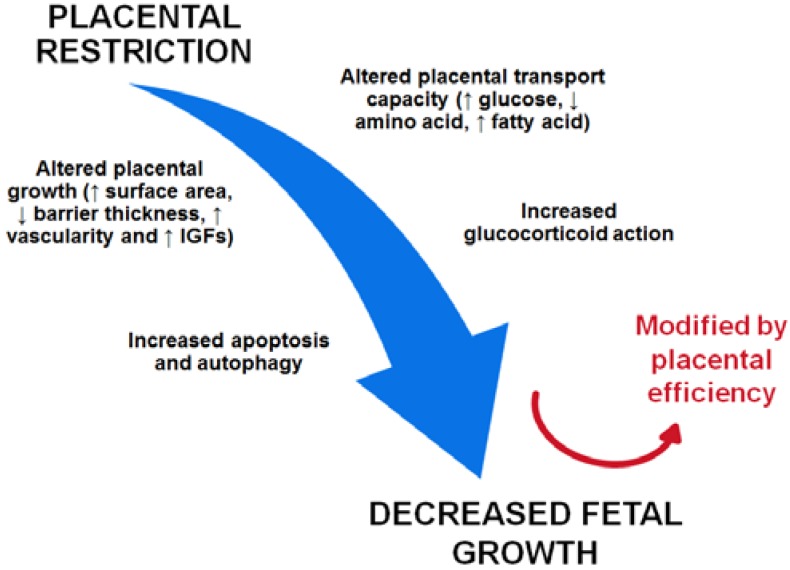
Summary of the placental adaptations that occur in the placental insufficiency-induced IUGR fetus and contribute to decreased fetal growth.

The placenta is the main interface between the fetus and mother. Placental insufficiency results in a failure of the placenta to deliver an adequate supply of substrates to the fetus and IUGR develops. A range of human and animal studies in IUGR pregnancies have suggested that placental restriction and insufficiency may result in a series of placental changes such as altered placental growth and placental substrate transport capacity, increased apoptosis and autophagy and increased glucocorticoid action (Figure 1). Such placental morphological and functional changes may consequently lead to decreased fetal growth and IUGR, which is modified by compensatory changes in placental efficiency. We have also noted that there are different patterns of placental changes associated with the human IUGR pregnancy and animal models of placental insufficiency induced IUGR. These differences may be dependent on the nature of the causes of IUGR, the time, duration and severity of insult exposure, species and *in vivo* or *in vitro* studies. While no model system completely encapsulates complete human IUGR, these model systems do allow us to carefully dissect aspects of the issue so as to further expand our understanding of the cellular and molecular mechanisms involved, and promote the development of therapeutic interventions.
